# First Results of an External Quality Assessment (EQA) Scheme for Molecular, Serological and Antigenic Diagnostic Test for SARS-CoV-2 Detection in Lombardy Region (Northern Italy), 2020–2022

**DOI:** 10.3390/diagnostics12061483

**Published:** 2022-06-16

**Authors:** Fabio Pasotti, Laura Pellegrinelli, Giuseppa Liga, Manuela Rizzetto, Giovanna Azzarà, Simona Da Molin, Oana Livia Lungu, Silvia Greco, Cristina Galli, Laura Bubba, Elena Pariani, Matteo Corradin, Danilo Cereda, Sabrina Buoro

**Affiliations:** 1 Centro di Riferimento per la Qualità dei Servizi di Medicina di Laboratorio di Regione Lombardia, 20162 Milano, Italy; fabio.pasotti@ospedaleniguarda.it (F.P.); giuseppa.liga@ospedaleniguarda.it (G.L.); manuela.rizzetto@ospedaleniguarda.it (M.R.); giovanna.azzara@ospedaleniguarda.it (G.A.); simona.damolin@ospedaleniguarda.it (S.D.M.); oana.lungu@ospedaleniguarda.it (O.L.L.); silvia.greco2@ospedaleniguarda.it (S.G.); sabrina_buoro@regione.lombardia.it (S.B.); 2 Dipartimento di Scienze Biomediche per la Salute, Università degli Studi di Milano, 20133 Milano, Italy; cristina.galli@unimi.it (C.G.); laurettabubba@gmail.com (L.B.); elena.pariani@unimi.it (E.P.); 3 Direzione Generale Welfare Regione Lombardia, 20124 Milano, Italy; matteo_corradin@regione.lombardia.it (M.C.); danilo_cereda@regione.lombardia.it (D.C.)

**Keywords:** SARS-CoV-2, external quality assessment (EQA), SARS-CoV-2 nucleic acid detection, anti-SARS-CoV-2 antibody test, SARS-CoV-2 direct antigens detection

## Abstract

For diagnosing SARS-CoV-2 infection and for monitoring its spread, the implementation of external quality assessment (EQA) schemes is mandatory to assess and ensure a standard quality according to national and international guidelines. Here, we present the results of the 2020, 2021, 2022 EQA schemes in Lombardy region for assessing the quality of the diagnostic laboratories involved in SARS-CoV-2 diagnosis. In the framework of the Quality Assurance Programs (QAPs), the routinely EQA schemes are managed by the regional reference centre for diagnostic laboratories quality (RRC-EQA) of the Lombardy region and are carried out by all the diagnostic laboratories. Three EQA programs were organized: (1) EQA of SARS-CoV-2 nucleic acid detection; (2) EQA of anti-SARS-CoV-2-antibody testing; (3) EQA of SARS-CoV-2 direct antigens detection. The percentage of concordance of 1938 molecular tests carried out within the SARS-CoV-2 nucleic acid detection EQA was 97.7%. The overall concordance of 1875 tests carried out within the anti-SARS-CoV-2 antibody EQA was 93.9% (79.6% for IgM). The overall concordance of 1495 tests carried out within the SARS-CoV-2 direct antigens detection EQA was 85% and it was negatively impacted by the results obtained by the analysis of weak positive samples. In conclusion, the EQA schemes for assessing the accuracy of SARS-CoV-2 diagnosis in the Lombardy region highlighted a suitable reproducibility and reliability of diagnostic assays, despite the heterogeneous landscape of SARS-CoV-2 tests and methods. Laboratory testing based on the detection of viral RNA in respiratory samples can be considered the gold standard for SARS-CoV-2 diagnosis.

## 1. Introduction

As SARS-CoV-2 spread globally becoming a pandemic, the urgent need to provide a laboratory diagnosis of the infection for clinical management of individual patients and for monitoring its spread in the community arose [1,2,3]. This resulted in a rapid development of diagnostic tests, including methods for the direct detection of the virus in biological samples (molecular and antigenic tests) and for indirect detection by documenting a contact with SARS-CoV-2 (serological antibodies tests). Molecular analyses on nasal-pharyngeal swabs (NPS) detecting the viral RNA are usually in use for SARS-CoV-2 diagnosis [4]. In particular, due to its accuracy and sensitivity, real-time RT-PCR is considered the gold standard method, being able to detect the viral RNA also in samples with low viral load [5,6,7]. However, faster methods based on the detection of the viral antigens are available [8]. Moreover, serological assays, able to determine a past infection through the identification of anti-viral specific antibodies, are complementary to the molecular tests, whenever a suspected COVID-19 case is not tested or results negative with molecular methods [9]. This approach is also useful to perform a retrospective diagnosis, to estimate SARS-CoV-2 time of infection, to measure the infection and exposure rates and to analyse the presence of neutralising antibodies after vaccination [10,11,12].

The impact of the pandemic on economic and social life required a rapid response able to face the health crisis that generated a huge number of diagnostic assays, which could have different sensitivity and specificity.

The diagnostic laboratories in Italy can implement the diagnostic assays of their choice after a proper validation with the gold standard methods. In order to guarantee a high quality in clinical diagnosis, it is required that the diagnostic laboratories participate at definite time to external quality assessments (EQA) consisting of a set of anonymised samples that must be analysed by each diagnostic laboratory. After matching the results obtained in each laboratory, the third party provides the evaluation of each diagnostic laboratory and shares the final report. The participation to EQA schemes is mandatory in Italy according to regional and national laws [13,14] and it is necessary to obtain and keep laboratory accreditation [12].

The regional reference centre for diagnostic laboratories quality (RRC-EQA) is in charge for the diagnostic laboratories’ evaluation through EQA schemes in Lombardy, reporting to the local health agencies any irregularity (i.e., no participation or non-compliant results) [15,16].

Here, we present the results of the 2020, 2021 and 2022 EQA schemes applied in Lombardy region in order to assess the quality of the laboratories operating in the region.

## 2. Materials and Methods

### 2.1. Organization of the EQA Schemes and Description of the Control Materials

In the framework of the Quality Assurance Programs (QAPs), the routinely EQA schemes are managed by the RRC-EQA of the Lombardy region and are usually carried out by all diagnostic laboratories. Regarding to the SARS-CoV-2 EQA schemes three EQA programs were organized. In particular:

(i). EQA of SARS-CoV-2 nucleic acid detection, represented by a coded panel consisting of 12 samples of control material (7 SARS-CoV-2 RNA positive and 5 negative) and analysed from July 2020 to July 2021;
(ii). EQA of anti-SARS-CoV-2 antibody test composed by a panel of 12 serum samples of control material (positive and negative depending on type of antibody) analysed from September 2020 to September 2021;
(iii). EQA of SARS-CoV-2 direct antigens detection consisting of a panel of 12 samples of control material (9 positive and 3 negative samples) analysed from April 2021 to April 2022.

Detailed information on the coding panel is shown in Tables. In detail, the control materials used in the EQA SARS-CoV-2 nucleic acid detection and direct antigen detection consisted of lyophilized cell culture supernatants obtained from SARS-CoV-2-positive/negative nasopharyngeal swabs. Each participating laboratory had to mix the lyophilized materials provided by the EQA with a dilution buffer or transport medium accordingly to the EQA instructions. The control material of the EQA anti-SARS-CoV-2-antibody detection consisted of a 0.5 mL vial of human plasma with known titre of IgG, IgM, IgA and total Ig. The classification of the samples and the concentration of SARS-CoV-2 was defined by Polymed s.r.l (Barberino Tavarnelle, Italy) in agreement with the RRC-EQA.

All samples were stored and shipped at 2–8 °C. Upon arrival, diagnostic laboratories were requested to store the samples at 2–8 °C until testing and to report the condition of the shipped samples by filling an online form at the RRC-EQA website (www.qualitalaboratorilombardia.it accessed on 20 April 2021). The diagnostic laboratories participating in the EQA were required to test the panels using each of their routine molecular and serological procedures and to report the results and the test carried out for performing the EQA on the RRC-EQA website (www.qualitalaboratorilombardia.it accessed on 20 April 2021). Data about nucleic acid extraction and detection methods, serological assays, instruments and reagents details, manufacturers, raw detection data, and qualitative results were required to be submitted. In particular, in the SARS-CoV-2 nucleic acid detection EQA the diagnostic laboratories were requested to specify the target gene of the assay (Nucleocapside, N; Spike, S; Envelope, E, ORF1ab, ORF8, RdRp) and to report qualitative value of the Cycle Threshold (Ct) of the run of the RT-PCR assay. In the EQA of anti-SARS-CoV-2 antibody testing, the diagnostic laboratories were requested to report qualitative and quantitative results of IgM, IgG, IgA and total Ig and to specify the type of antibodies (anti-N, S1, S2, S1/S2, S1-RBD, Trimeric S). As concern the EQA of SARS-CoV-2 direct antigens detection, the diagnostic laboratories were requested to report qualitative and quantitative results and to specify the viral antigen targeted by the test (N and/or S).

For molecular detection of SARS-CoV-2, before participating in the regional EQA, each laboratory was requested to participate to a preliminary assessment to evaluate the concordance among different commercial assays by testing a panel of 15 samples (5 SARS-CoV-2 RNA-positive and 10 negative samples) [16] to those obtained by the reference centre for COVID-19 virological surveillance at the Department of Biomedical Sciences for Health, University of Milan [17], in the Lombardy region.

The final report was distributed within seven working days from each EQA scheme deadline.

### 2.2. Analysis of the Results of SARS-CoV-2 Nucleic Acid Detection EQA

The data obtained from each diagnostic laboratory for the SARS-CoV-2 nucleic acid detection EQA were reported overall as number of participating laboratories reporting data. Data were collided by assay in use and by target. The overall number of tests performed was also included in the analysis. Based on the Ct value obtained, each sample was reported as positive, weak positive, negative (when the Ct value was under the cut-off of the assay in use) or invalid (when the test failed for the absence of internal control).

### 2.3. Analysis of the Results of Anti-SARS-CoV-2 Antibody Testing EQA

The descriptive analysis of the anti-SARS-CoV-2 antibody EQA was performed. The number of the diagnostic laboratories that reported the results, the number of the different serological antigenic assays and the number of tests performed were reported. Per each serological assay the results were analysed by target and reported as negative (i.e., under the cut-off), undetermined (i.e., when the result obtained was not conclusive) or positive (i.e., over the methods’ cut-off).

### 2.4. Analysis of the Results EQA of SARS-CoV-2 Direct Antigens Detection EQA

The descriptive analysis of the EQA of SARS-CoV-2 direct antigens detection results was performed. The number of the diagnostic laboratories that reported the results, the number of the different serological antigenic assays and the number of tests performed were reported. Per each assay the result was reported as negative (i.e., under the cut-off), undetermined (i.e., when the result obtained was not conclusive) or positive (i.e., over the methods’ cut-off).

## 3. Results

### 3.1. SARS-CoV-2 Nucleic Acid Detection EQA

Table 1 reports the number of laboratories participating to each quality assurance exercise, the number of assays used, the number of reported results of the SARS-CoV-2 nucleic acid detection EQA program that included 12 samples distributed from July 2020 to July 2021. Over the time, the number of laboratories participating in this EQA changed from 56 on July 2020 to 86 on July 2021 that carried out from 57 (sample #1) to 115 (sample #12) assays, resulting in 1938 molecular tests (Table 1).

The percentage of concordance of 1938 tests carried out within the SARS-CoV-2 nucleic acid detection EQA was 97.7%. The value of concordance rose to 99.8% by ruling out from the analysis all the “invalid” results (i.e., inconclusive results). Considering results of the positive samples only, the concordance was 99.7% and when weak positive samples (samples #3 and #5) were excluded from the analysis the concordance was 99.9% (Table 1).

The concordance of negative samples #2 and #8 was 100%, of sample #10 was 99% and was 79.4% for the negative sample #4. The analysis of the negative sample #6 gave an invalid result for 3.5% of the diagnostic assays (Table 1).

### 3.2. Anti-SARS-CoV-2 Antibody Testing EQA

Table 2 reports the number of laboratories reporting, the number of assays, the number of reported testing and results of the anti-SARS-CoV-2 antibody EQA program carried out from September 2020 to September 2021 and including 12 quality exercises. According to the reported results, the most frequently used test was that for the detection of IgG that was conducted by 66 laboratories for sample #1 and by 77 laboratories for sample #12, with 33 different assays to test sample #11. The test for the detection of IgA was carried out only by two laboratories for sample #1 and by one laboratory for samples #3, #4 and #5. Overall, 1875 tests were carried out within the anti-SARS-CoV-2 antibody EQA: in detail, 845 tests targeted IgG, 450 tests targeted IgM, 5 tests identified IgA and 389 tests assayed the presence of total Ig (Table 2). The different number of tests for specific immunoglobulins overlapped the clinical utility of these tests [18]. Overall, the samples of this EQA were investigated with a number of assays ranging from 2 to 33 (Table 2). The overall concordance was 93.9%; in particular, the concordance for the negative samples was 99.1% for IgG, 89% for IgM, 100% for IgA and 100% for total Ig. For positive samples, the concordance was 99.6% for IgG, 100% for IgA and 93.8% for total Ig (Table 2); the concordance of the IgM testing was 79.6%, this latter percentage may depend on the fact that—for a number of assays—the value of positive samples was close to the cut-off level of the assay. Samples #11 and #12 were from COVID-19-vaccinated patients and these samples did not allow a positive response for those analytical tests that target antigens other than the viral protein S.

### 3.3. SARS-CoV-2 Direct Antigens Detection EQA

Table 3 reports the number of laboratories reporting, the number of assays, the number of reported testing, the classification of the samples and results of the of SARS-CoV-2 direct antigens detection EQA program carried out from April 2021 to April 2022 and including 12 samples. Overall, all samples of this EQA were investigated with a number of assays ranging from 27 (for sample #2) to 35 (for sample #5) (Table 3). The overall concordance of 1495 tests carried out within the SARS-CoV-2 direct antigens detection EQA was 85%. The concordance was 97.8% for positive samples (samples #1, #5, #6, #9, #10), 99.2% for negative samples (samples #2, #4 and #8) and 64.7% for weak positive (borderline) samples (samples #3, #7, #11 and #12). In the case of samples #2 and #7, 1.6% and 1.7%, respectively, of the results were reported as “invalid” since problems in storage and handling of these samples were reported from several laboratories.

## 4. Discussion

In laboratory medicine EQA programs are pivotal to assess the performance and status of diagnostic assays in clinical laboratories [19]. Reproducibility and reliability of diagnostic assays are of particular importance in the clinical management and for public health purpose [19]. Laboratories should perform verification studies before routine implementation of novel tests and monitor reliability throughout the entire process through routinely quality management [12]. Moreover, the inter-laboratory comparison allows to evaluate participants’ performance by appraising the analytical performance and test interpretation and to evaluate method performance.

The results of the EQA schemes here presented were organized in 2020, 2021 and 2022 to evaluate the accuracy—as well as the ability to detect positive and negative samples in clinical practice—of SARS-CoV-2 diagnostic assays among clinical laboratories in the Lombardy region. Three EQAs were organized: an EQA of SARS-CoV-2 nucleic acid detection was conducted since July 2020, an EQA of anti-SARS-CoV-2 antibody testing was conducted since September 2020 and an EQA of SARS-CoV-2 direct antigens identification was carried out since April 2021. The laboratories participating in these EQA programs were required to assay the panels using their routine procedures and report their qualitative and/or quantitative results.

A level of concordance below 100% could represent a problem in a pandemic situation and in the control of SARS-CoV-2 spread due to the number of false negative results. In our experience, the percentage of concordance of 1938 tests carried out within the SARS-CoV-2 nucleic acid detection EQA was 97.7%, which can depend on different cut-off as defined by Ct values or algorithm to consider a result conclusive or inconclusive in the performing laboratory. In fact, the concordance rises to 99.8% by ruling out from the analysis all the “invalid” results (i.e., inconclusive results). Some of the SARS-CoV-2 RT-PCR kits did not detect the internal control of the negative sample #4, making the value of concordance for this sample equal to 79.4%; this could depend on a low concentration of human cells.

The overall concordance of 1875 tests carried out in the anti-SARS-CoV-2 antibody testing EQA was 93.9%; in particular, the concordance for the positive samples reached 99.6% for IgG, 100% for IgA and 93.8% for total Ig, but the concordance for IgM was 79.6%. The lower level of concordance for IgM could depend on the fact that, for a number of commercial assays, the value of IgM was close to the cut-off level of the assay. As already reported in the recent literature [20,21], immunochromatographic tests—although scarcely used by the diagnostic laboratories involved in this EQA program [3]—have showed a low level of concordance, thus their implementation should be considered critical in clinical setting. Serum samples #11 and #12 were from COVID-19-vaccinated patients and the particular type of sample examined did not allow a positive response for those analytical tests that target antigens other than the viral S protein.

The level of concordance of 1495 tests carried out in the EQA of SARS-CoV-2 direct antigens detection was 85%, thus achieving the World Health Organization (WHO) criteria for the use of antigenic test (minimum performance requirement ≥ 80%) [22]. The lowest level of concordance was reported for samples #3, #7, #11 and #12 that all were weak positive (borderline) samples; this could mean either that the available commercial kits have a different level of sensibility or that the results in the case of a weak positive sample could be laboratory-dependent. Both aspects could heavily impact on SARS-CoV-2 spread in consideration that SARS-CoV-2 direct antigens detection may be used in screening programs. The evidence from this study are in agreement with the findings of Jeulien et al. [8] who have determined that high value of sensitivity of direct antigenic tests can be obtained only for SARS-CoV-2 positive samples with a Ct value ≤ 25 and that in case of asymptomatic patients or individuals with low viral load the sensitivity was of 58.1% [8].

## 5. Conclusions

In conclusion, EQA schemes are of crucial importance to assess the diagnostic performance of individual laboratories and to improve the overall quality. The EQA schemes here designed by the RRC-EQA for assessing the clinical accuracy of SARS-CoV-2 diagnosis in the Lombardy region highlighted a suitable reproducibility and reliability of diagnostic assays, despite the heterogeneous landscape of SARS-CoV-2 testing and methods. Results from this study show that the measurement of IgM anti-SARS-CoV-2 and the direct detection of SARS-CoV-2 antigens in case of weak positive samples have the lowest level of concordance and that the laboratory testing based on the detection of viral RNA can be considered the gold standard for SARS-CoV-2 diagnosis.

## Figures and Tables

**Table 1:** Number of laboratories (lab.) reporting, number of assays, number of reported testing and results of the SARS-CoV-2 nucleic acid detection EQA.

# Sample	No. of Lab. Reporting	No. of Different Assays	No. of Tests	Classification	Invalid (%)	Negative (%)	Weak Positive (%)	Positive (%)
1	56	57	119	Positive	0	0	0	100
2	56	62	119	Negative	0	100	0	0
3	61	65	129	Weak Positive	0	0	0.8	99.2
4	61	72	136	Negative	20.6	79.4	0	0
5	63	76	140	Weak Positive	0	0	0.7	99.3
6	66	78	144	Negative	3.5	96.5	0	0
7	69	91	158	Positive	0	0	0	100
8	74	95	166	Negative	0	100	0	0
9	79	121	199	Positive	0	0.5	0	99.5
10	81	120	204	Negative	1	99	0	0
11	82	122	209	Positive	0	0	0	100
12	86	115	215	Positive	0	0	0	100

**Table 2:** Number of laboratories reporting, number of assays, number of reported testing and results of the anti-SARS-CoV-2 antibody EQA.

# Sample	Target	Classification	No. of Lab Reporting	No. of Different Assays	No. of Testing	Total Results		
						Negative (%)	Doubt (%)	Positive (%)
1	IgG	Positive	66	17	81	0	1.2	98.8
	IgM	Positive	30	14	33	30.3	3.0	66.7
	IgA	Positive	2	2	2	0	0	100
	Total Ig	Positive	30	4	30	0	0	100
2	IgG	Negative	69	18	83	100	0	0
	IgM	Negative	33	14	36	100	0	0
	IgA	Negative	2	2	2	100	0	0
	Total Ig	Negative	31	5	31	100	0	0
3	IgG	Positive	69	19	77	1.3	0	98.7
	IgM	Positive	38	16	40	30.0	2.5	67.5
	IgA	Positive	1	1	1	0	0	100
	Total Ig	Positive	32	5	32	0	0	100
4	IgG	Negative	69	19	75	97.3	0	2.7
	IgM	Negative	39	16	40	100	0	0
	IgA	Negative	1	1	1	100	0	0
	Total Ig	Negative	34	6	34	100	0	0
5	IgG	Positive	69	20	75	98.7	1.3	0
	IgM	Positive	43	16	43	16.3	0	83.7
	IgA	Positive	1	1	1	100	0	0
	Total Ig	Positive	33	7	34	100	0	0
6	IgG	Negative	69	27	77	100	0	0
	IgM	Negative	42	15	42	100	0	0
	IgA	/	/	/	/	/	/	/
	Total Ig	Negative	31	8	34	100	0	0
7	IgG	Positive	71	27	82	0	0	100
	IgM	Positive	40	13	41	36.6	2.4	61
	IgA	/	/	/	/	/	/	/
	Total Ig	Positive	32	7	37	2.7	0	94.6
8	IgG	Positive	72	30	84	0	0	100
	IgM	Positive	39	13	40	12.5	0	87.5
	IgA	/	/	/	/	/	/	/
	Total immunoglobulins	Positive	31	6	36	0	0	100
9	IgG	Positive	72	29	84	0	0	100
	IgM	Negative	38	12	39	94.9	0	5.1
	IgA	/	/	/	/	/	/	/
	Total Ig	Positive	32	6	38	0	0	100
10	IgG	Positive	72	30	84	0	0	100
	IgM	Positive	37	11	38	2.6	2.6	94.7
	IgA	/	/	/	/	/	/	/
	Total Ig	Positive	35	7	41	0	0	100
11	IgG	Positive	70	33	83	0	0	100
	IgM	Negative	37	11	38	52.6	2.6	44.7
	IgA	/	/	/	/	/	/	/
	Total Ig	Positive	35	7	42	0	0	100
12	IgG	Positive	77	32	86	12.8	0	87.2
	IgM	Negative	34	11	38	13.2	0	86.8
	IgA	/	/	/	/	/	/	/
	Total Ig	Positive	33	8	40	50	0	50

**Table 3:** Number of laboratories reporting, number of assays, number of reported testing and results of SARS-CoV-2 direct antigens detection EQA.

# Sample	Classification	No. of Lab Reporting	No. of Different Assays	No. of Testing	Total Results			
					Negative (%)	Doubt (%)	Positive (%)	Invalid (%)
1	Positive	93	30	125	2.4	0.0	97.6	0.0
2	Negative	94	27	124	98.4	0.0	0.0	1.6
3	Weak Positive (borderline)	95	30	123	32.5	4.1	63.4	0.0
4	Negative	97	33	126	99.2	0.8	0.0	0.0
5	Positive	91	32	121	0.8	0.0	99.2	0.0
6	Positive	96	33	122	2.5	0.0	97.5	0.0
7	Weak Positive (borderline)	94	31	119	24.4	9.2	64.7	1.7
8	Negative	95	35	121	100.0	0.0	0.0	0.0
9	Positive	96	33	122	22.1	9.0	68.9	0.0
10	Positive	103	34	130	0.0	0.0	100	0.0
11	Weak Positive (borderline)	103	33	132	65.2	5.3	29.5	0.0
12	Weak Positive (borderline)	103	34	130	30.8	3.8	65.4	0.0

## Data Availability

Not applicable.
